# The AMP-Dependent Protein Kinase (AMPK) Activator A-769662 Causes Arterial Relaxation by Reducing Cytosolic Free Calcium Independently of an Increase in AMPK Phosphorylation

**DOI:** 10.3389/fphar.2017.00756

**Published:** 2017-10-18

**Authors:** Yi Huang, Corey A. Smith, Grace Chen, Bharti Sharma, Amy S. Miner, Robert W. Barbee, Paul H. Ratz

**Affiliations:** ^1^Department of Emergency Medicine and Physiology, Virginia Commonwealth University, Richmond, VA, United States; ^2^Department of Biochemistry and Molecular Biology, School of Medicine, Virginia Commonwealth University, Richmond, VA, United States

**Keywords:** vascular smooth muscle, cell signaling, myosin, rhoA, MYPT1, simvastatin, metformin, berberine

## Abstract

Although recent studies reveal that activation of the metabolic and Ca^2+^ sensor AMPK strongly inhibits smooth muscle contraction, there is a paucity of information about the potential linkage between pharmacological AMPK activation and vascular smooth muscle (VSM) contraction regulation. Our aim was to test the general hypothesis that the allosteric AMPK activator A-769662 causes VSM relaxation via inhibition of contractile protein activation, and to specifically determine which activation mechanism(s) is(are) affected. The ability of A-769662 to cause endothelium-independent relaxation of contractions induced by several contractile stimuli was examined in large and small musculocutaneous and visceral rabbit arteries. For comparison, the structurally dissimilar AMPK activators MET, SIM, and BBR were assessed. A-769662 displayed artery- and agonist-dependent differential inhibitory activities that depended on artery size and location. A-769662 did not increase AMPK-pT172 levels, but did increase phosphorylation of the downstream AMPK substrate, acetyl-CoA carboxylase (ACC). A-769662 did not inhibit basal phosphorylation levels of several contractile protein regulatory proteins, and did not alter the activation state of rhoA. A-769662 did not inhibit Ca^2+^- and GTPγS-induced contractions in β-escin-permeabilized muscle, suggesting that A-769662 must act by inhibiting Ca^2+^ signaling. In intact artery, A-769662 immediately reduced basal intracellular free calcium ([Ca^2+^]_i_), inhibited a stimulus-induced increase in [Ca^2+^]_i_, and inhibited a cyclopiazonic acid (CPA)-induced contraction. MET increased AMPK-pT172, and caused neither inhibition of contraction nor inhibition of [Ca^2+^]_i_. Together, these data support the hypothesis that the differential inhibition of stimulus-induced arterial contractions by A-769662 was due to selective inhibition of a Ca^2+^ mobilization pathway, possibly involving CPA-dependent Ca^2+^ entry via an AMPK-independent pathway. That MET activated AMPK without causing arterial relaxation suggests that AMPK activation does not necessarily cause VSM relaxation.

## Introduction

Contraction of VSM is highly dependent on the degree of myosin light chain (MLC) phosphorylation which, in turn, is dependent on an elevation in [Ca^2+^]_i_ leading to increases in MLC kinase (MLCK) activity, and on rhoA kinase (ROCK)- and PKC- dependent inhibition of MLC phosphatase activity (Guibert et al., 2008; Puetz et al., 2009). RhoA-dependent regulation of actin polymerization also appears to play a role (Pritchard et al., 2004; Dai et al., 2008). Notably, AMPK has been shown to cause endothelium-independent arterial relaxation (Rubin et al., 2005; Goirand et al., 2007) by inhibiting all of these mechanisms. For example, AMPK can inhibit MLCK (Horman et al., 2008), rhoA (Gayard et al., 2011), rhoA kinase (ROCK) (Wang et al., 2011), and PKC (Davis et al., 2012), and can activate K^+^ channels (Schneider et al., 2015) that would reduce membrane excitation, and activate the sarcoplasmic/endoplasmic reticulum Ca^2+^ ATPase (SERCA) pump that would enhance Ca^2+^ sequestration, lowering [Ca^2+^]_i_ (Schneider et al., 2015). Notably, small arteries (i.e., mouse saphenous artery) appear to respond less well than large arteries (i.e., mouse aorta) (Davis et al., 2012), suggesting heterogeneity of VSM responsiveness to AMPK within different regions of the vascular tree. Arteries along the vascular tree can be divided into large elastic (e.g., aorta and carotid), large muscular (e.g., femoral and renal), small muscular (e.g., branching arteries such as second to fourth order mesenteric and epigastric) and the still smaller arterioles (Ratz, 2016). Whereas conduit and larger muscular arteries responsible for “buffering” pressure pulses generally respond to stimuli with strong, tonic contractions that enter the latch state (Murphy, 1988), small muscular arteries and arterioles responsible for blood flow regulation generally respond in a biphasic manner with a strong initial contraction that fades to a weaker sustained response or that undergoes rhythmic tone. Moreover, different segments of the arterial tree display some differences in their responsiveness to the major VSM stimuli (e.g., α-adrenergic stimuli, Ang II, thromboxane and VP) (Ratz, 2016). No study has yet compared the effects of A-769662 in large and small muscular arteries that display functionally distinct responses to contractile stimuli.

Pharmacologically, the thienopyridone derivative A-769662 can selectively bind the AMPK β1 regulatory subunit and allosterically elevate the kinase activity of the AMPK α subunit (Scott et al., 2008; Guigas et al., 2009; Ducommun et al., 2014; Timmermans et al., 2014; Viollet et al., 2014). Generally, stimuli that activate AMPK do so via increasing AMPK phosphorylation. However, A-769662 can cause an increase in phosphorylation of the AMPK downstream target acetyl-CoA carboxylase (ACC) without generating an increase in AMPK-pT172 above the basal level (Goransson et al., 2007; Foretz et al., 2010), and A-769662 has been shown to cause VSM relaxation (Schneider et al., 2015), but the precise mechanism remains to be determined. The present study was designed to quantify the relative ability of A-769662 to increase AMPK activity and inhibit contractions *in vitro* in rabbit large and small musculocutaneous and visceral muscular arteries. Specifically, the aims were to (1) determine whether A-769662 inhibits contractions induced by all of the major VSM contractile stimuli, and thus, can be classified as a general VSM relaxant agent, (2) compare the ability of A-769662 and other known AMPK activators to cause increases in AMPK-pT172, an index of AMPK activation, and (3) identify the mechanism(s) by which A-769662 causes VSM relaxation.

## Materials and Methods

### Animals

All studies were approved by the Institutional Animal Care and Use Committee of Virginia Commonwealth University and conform to the Public Health Service Policy on Humane Care and Use of Laboratory Animals (2015) and the National Research Council “Guide for the Care and Use of Laboratory Animals” (Eighth Edition). Specific-pathogen free, male, New Zealand White rabbits (weight range: 2.8–3.8 kg, mean weight: 3.35 ± 0.23 kg, age range: 12–15 weeks) were obtained from Robinson Services, Inc., and maintained in the vivarium at 19-22°C and a 12 h light, 12 h dark cycle for at least 6–7 days prior to experimentation. Animals were individually housed, provided environmental enrichment and fed a combination of pelleted high-fiber rabbit food (Harlan Teklad 2031, ∼1 cup/day) and hay. Specific-pathogen free mice were obtained from Jackson Laboratories and maintained in the vivarium at 22–23°C and a 12 h light, 12 h dark cycle for at least 3–4 days prior to experimentation. Mice (average weights ∼23–30 g) were normally group housed (except for aggressive male mice, which were individually housed with added enrichment) and fed Envigo Teklad 7012 Rodent Diet *ad lib*.

### Tissue Preparation

Large (FA) and small (superficial inferior EA) muscular arteries perfusing musculocutaneous vascular beds, and large (RA) and small (third order MA) muscular arteries perfusing the viscera, were isolated from rabbits and prepared as described previously with minor modifications (Ratz, 1990). Also isolated were the rabbit carotid artery (CA), rabbit liver, mouse MA, mouse ileum and mouse extensor digitorum longus. Arteries were cleaned of adhering tissues by microdissection (Olympus SZX12) and cut into rings ∼2.5 mm wide. The liver was perfused with cold (∼4°C) saline prior to removal to clear the vascular bed of blood and quickly cool cells, and sliced into ∼0.5 mm thin sheets. Tissues were placed in a normal physiological salt solution (NPSS), the composition of which was, in mM, 140 NaCl, 4.7 KCl, 1.2 Na_2_HPO_4_-7H_2_O, 2.0 MOPS, 0.02 Na_2_ethylenediamine tetraacetic acid to chelate heavy metals, 5.6 D-glucose, 1.6 CaCl_2_ and 1.2 MgCl_2_, made with high-purity (17 MΩ) deionized water and adjusted using NaOH to a pH of 7.4 at 37°C. Each artery ring was either not stretched (retained at zero external force, F_ze_) or secured in a tissue myograph (Model 610M, Danish Myo Technology) and adjusted to the optimum length for muscle contraction (L_0_) using an abbreviated length-tension protocol in which tissues were contracted by addition of a physiological salt solution adjusted so that the KCl concentration would rapidly produce a maximum contraction (KPSS; 110 μM KCl substituted isosmotically in the NPSS for NaCl) (Ratz and Murphy, 1987; Ratz, 1993). For each tissue, the maximum value of the KPSS-induced contraction at L_0_ was recorded as the F_0_ value, and all subsequent contractile responses were normalized to this value. Thus, the strength of a subsequent response to a contractile stimulus was reported as the ratio F/F_0_, where F_0_ was the KPSS induced response made equal to the value “1.” Unless otherwise indicated, all experiments were conducted on denuded arteries in the presence of 100 μM L-NMMA and 30 μM ODQ to block vasodilatation due to nitric oxide release and activation of guanylyl cyclase.

### Concentration-Response Curve (CRC) Protocol

Artery rings at L_0_ were contracted by the step-wise, cumulative addition of increasing concentrations of one of the following five contractile agonists: Ang II, KCl (as KPSS), PE, U-46619 or VP. Rabbit arteries were used for all CRC analyses except for the effect of A-769662 (Tocri, Bio-Techne Corp., Minneapolis, MN, United States) on U-46619. Rabbit MA does not respond to Ang II or VP, and responds poorly to U-46619 (Ratz, 2016), whereas mouse MA responds strongly to U-46619. Thus, when considering MA, no data (**Figures 3D,T**, n.d.) was obtained for Ang II and VP CRCs, and mouse MA was used to assess the effect of A-769662 on a U-46619 CRC (**Figure 3P**). Permeabilized tissues were activated by addition of Ca^2+^ and GTPγS as described previously (Clelland et al., 2011). Data were reported as active force normalized to F_0_ (F/F_0_), and thus, an F/F_0_ value of 1 was equal to the maximum KPSS-induced contraction achieved during the initial determination of L_0_.

### Chemical Permeabilization

**FIGURE 1:**
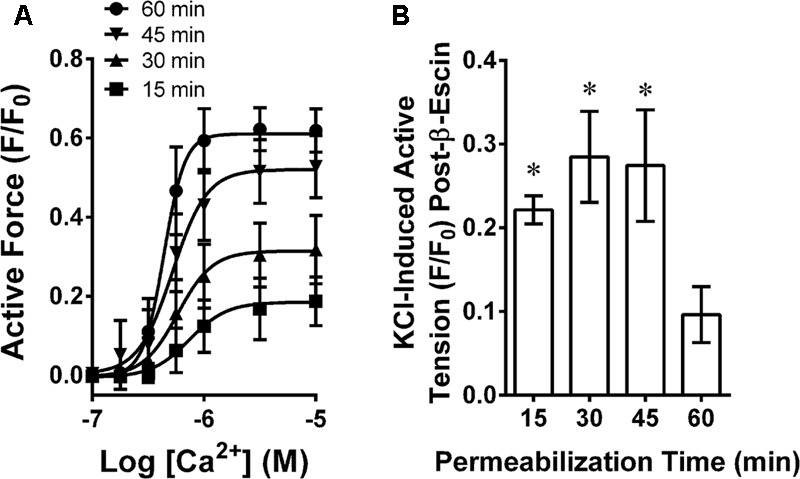
Determination of optimum β-escin permeabilization time for MA showing a Ca^2+^ concentration response curve **(A)** and time-dependent loss of plasma membrane-dependent contraction **(B)**. Data are mean values ± SE; *n* = 4. ^∗^ is *P* < 0.05 (one-sample *t*-test) compared to zero.

Rabbit MA was chemically permeabilized with β-escin as described previously (Clelland et al., 2011) with minor modifications. In short, each artery ring at L_0_ was incubated at 30°C in a Ca^2+^-free “relaxing solution” (in mM: 74.1 potassium methanesulfonate, 4.0 magnesium methanesulfonate, 4.0 Na2ATP, 4.0 EGTA, 5.0 creatine phosphate, 30.0 PIPES, adjusted to pH 7.1 with 1 N KOH and ionic strength 180 with additional 0.5 M potassium methanesulfonate). Permeabilization was achieved by incubating tissues in β-escin (Sigma–Aldrich, St. Louis, MO, United States) dissolved in Ca^2+^-free relaxing solution at room temperature. To identify the optimum permeabilization time, tissues were exposed to 100 μM β-escin for 15, 30, 45 and 60 min, then subjected to a Ca^2+^ CRC (**Figure 1A**) and, after washout in a Ca^2+^-free relaxing solution, exposed to KPSS (**Figure 1B**). The relative responsiveness to Ca^2+^ compared to KPSS is an index of the fraction of cells effectively permeabilized. Exposure for 60 min was required to produce a strong and potent Ca^2+^ CRC response with a negligible residual response to KPSS (not different than zero, *P* < 0.05, **Figure 1**). Thus, subsequent tissues were permeabilized with 100 μM β-escin for 60 min. Following permeabilization, each tissue was washed thoroughly with Ca^2+^-free relaxing solution to remove β-escin. The calcium ionophore A23187 (10 μM) was added to deplete sarcoplasmic reticulum Ca^2+^, and 1 unit/ml of calmodulin (Sigma–Aldrich, St. Louis, MO, United States) was added to compensate for loss during permeabilization. All permeabilized tissues and solutions were maintained at room temperature.

### Phosphoprotein Analysis

The ability of A-769662 to activate AMPK, and to alter phosphorylation of the downstream effectors ACC, MLC, rhoA, cofilin, myosin phosphatase regulatory subunit (MYPT1), and phospholamban (PLB), was assessed by standard 1-dimensional sodium dodecylsulfate gel electrophoresis followed by “Western” blotting, enhanced chemiluminescence (Pierce, Thermo Fisher Scientific Inc., Rockford, IL, United States) and image analysis of exposed x-ray film (CLINICAELECT Blue, Carestream Health, Inc., Rochester, NY, United States) using an imager (Konica Minolta Medical & Graphic, Inc.) and image analysis software (Image J^1^), as described previously (Ratz, 2001) with minor modifications. The degree of change in phosphorylation was normalized to the basal (control) phosphorylation level, and the data were presented as fold-control, unless otherwise indicated. At the appropriate time point, tissues were rapidly frozen in dry ice-cooled acetone containing 6% trichloroacetic acid, 10 μM dithiothreitol and 30 mM NaF, slowly thawed, dried, weighed and homogenized with a buffer containing 25 mM Tris- Base, 20 mM dithiothreitol, 10% glycerol, 1% sodium dodecylsulfate, 5 mM EGTA, 1 mM EDTA, 50 mM NaF, 1 mM activated Na^+^ orthovanadate, 10 μg/ml leupeptin, 10 μg/ml aprotonin and 1 mM AEBSF. Protein samples (15–20 μg/μl) resolved by gel electrophoresis and Western-blotted onto polyvinylidene difluoride membranes were probed using anti-phospho AMPK (pT172) antibody (1:1000, Cell Signaling Technology, United States), anti-phospho ACC (pS79) antibody (1:1000, Cell Signaling Technology, United States), anti-phospho cofilin (pS3) antibody (1:500, Cell Signaling Technology, United States), anti-phospho MLC (pS19) antibody (1:1000, Sigma–Aldrich, United States), anti-phospho MYPT1 (T853) antibody (1:1000, EMD Millipore, United States), anti-phospho PLB (pS16) antibody (1:1000, Badrilla, United Kingdom), and anti-phospho rhoA (pS188) antibody (1:500, Santa Cruz Biotechnology, United States) at 4°C overnight. Goat-α-rabbit IgG- horseradish peroxidase was used as secondary antibody (1:2000, Santa Cruz Biotechnology, United States) at room temperature for 1 h. All lanes were loaded with identical quantities of protein. However, two proteins expressed by housekeeping genes, β-actin and GAPDH, were routinely monitored to assess uniform protein loading across lanes. Data that did not display the expected equivalent protein loading were discarded. For these proteins, blots were incubated with β-actin antibody (C4) horseradish peroxidase (1:100000, Santa Cruz, CA, United States) and GADPH antibody (1:5000, Santa Cruz, CA, United States) at room temperature for 1 h.

#### Intracellular Free Calcium

Intracellular free calcium concentration was measured as previously described (Ratz, 1993) with minor modifications. Tissues at L_0_ in an aerated muscle chamber designed for microscopic imaging (Danish Myo Technology) were placed on the stage of an inverted microscope (Olympus IX71) and loaded for 2.5 h with 7.5 μM fura 2-PE3 (AM) and 0.01% (wt/vol) Pluronic F-127 (TefLabs, Austin, TX, United States) to enhance solubility. Fluorescence emission at 510 nm was collected by a photomultiplier tube for excitations at 340 and 380 nm (DeltaRam V, Photon Technologies, Lawrenceville, NJ, United States), and emission intensities were expressed as 340/380 nm ratios with the use of Felix software (Photon Technology International). Raw ratios prior to background subtraction were reported as “arbitrary units.” Background fluorescence determined by incubating tissues in 4 mM MnCl_2_ plus 30 μM ionomycin was subtracted from all 340 and 380 nm signals before calculation of the 340/380 nm fluorescence ratio. For accuracy, each tissue served as its own control. Each tissue was contracted with 1 μM PE until a peak contraction was produced (control responses), washed twice to remove PE and permit relaxation (∼25 min), treated for ∼25 min with a drug, and contracted a second time with 1 μM PE. Calcium and force responses produced during exposure to a drug were reported as a ratio of the peak responses in the presence of drug divided by the peak responses in the absence of drug (control).

### Additional Drugs, Data Analysis and Statistics

In some experiments, the natural compound BBR, anti-atherogenic drug SIM, and anti-diabetic drug MET were used for comparison with A-769662 because these agents are reported also to activate AMPK (Ewart and Kennedy, 2011; Hardie, 2013). In the chemical permeabilization experiment, the Ca^2+^-calmodulin-MLC kinase inhibitor trifluoperazine (TFP), and ROCK inhibitor HA-1077, were used for comparison with A-769662. Protein kinase A (PKA) and protein kinase G (PKG) activation are well-known to cause VSM relaxation. Thus, for a comparison, forskolin (FSK) was used to activate adenylyl cyclase and, thus, cAMP and PKA, and 8-bromo-cGMP (8b-cGMP) was used to activate PKG. Cyclopiazonic acid (CPA) was used to inhibit the sarcoplasmic and endoplasmic reticulum Ca^2+^-ATPase (SERCA), and 3 μM YM-58483 (a.k.a., BTP-2), was used to block store-operated Ca^2+^ channels (Harper and Poole, 2011) because this drug displays marked selectivity for inhibition of store-operated over non-selective Ca^2+^ channels. Moreover, YM-58483 selectively inhibits TRPC channels over TRPV channels, two proteins implicated in forming store-operated Ca^2+^ channels (He et al., 2005). All data were analyzed using Graph Pad Prism 6.0 software (GraphPad Software, Inc., La Jolla, CA, United States) and are presented as mean ± standard error of the mean (SE). CRC data for each tissue and each contractile agonist were fitted to a sigmoidal curve (Kenakin, 1997), except for tissues strongly inhibited by SIM, where force values at each agonist concentration were compared. For statistical analyses, and as indicated in the figure legends, data (“test” and “control”) were evaluated by the One-Sample *t*-test, Student’s *t*-test or Analysis of Variance (ANOVA) and the Dunnett’s *post hoc* test, and the Null hypothesis was rejected at *P* < 0.05. When applying the *t*-test two times, the Bonferroni correction was applied.

## Results

### Expression of α and β AMPK Subunits in Rabbit Arteries

**FIGURE 2:**
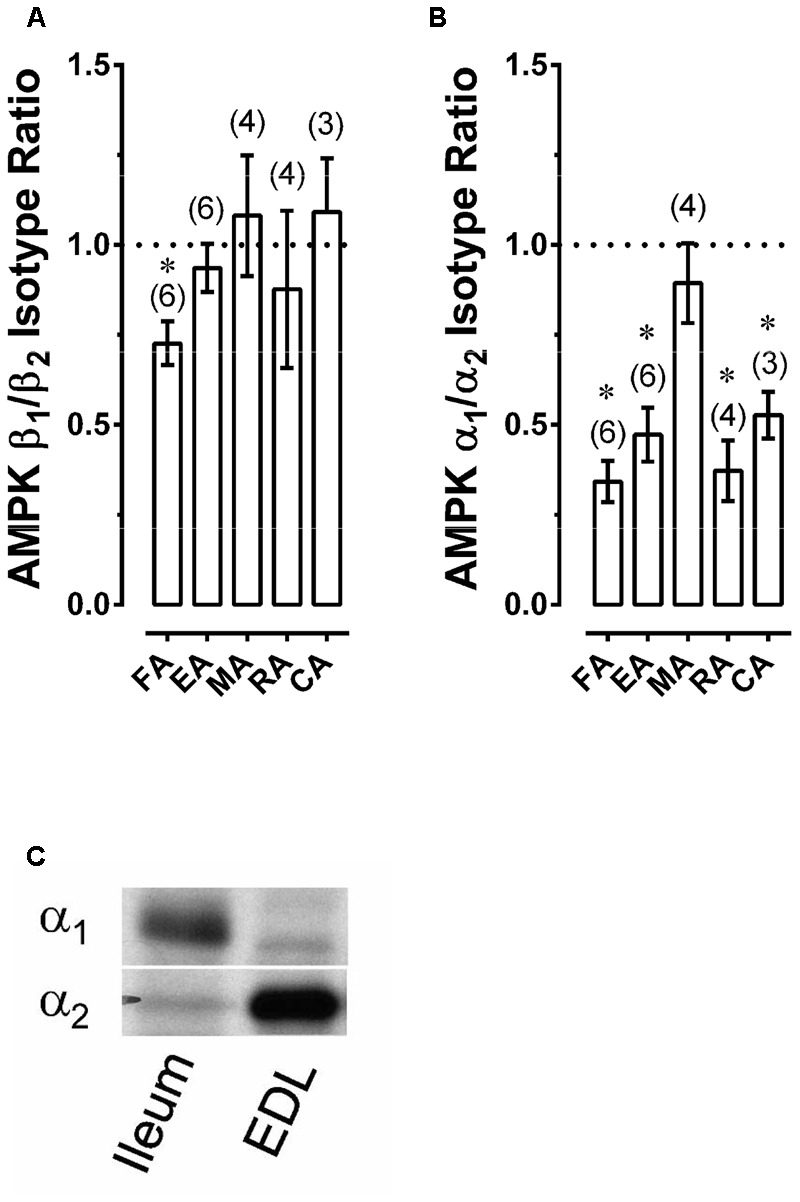
Western blot data revealing AMPK β_1_/β_2_ **(A)** and α_1_/α_2_ **(B)** expression ratios for selected rabbit arteries, as well as an example revealing that use of a Western blot ratiometric α_1_/α_2_-AMPK analysis was capable of detecting the expected high α_1_/α_2_ ratio in mouse ileum and low α_1_/α_2_ ratio in mouse extensor digitorum longus (EDL) **(C)**. Data in **(A,B)** are means ± SE; *n* values are in parenthesis; ^∗^*P* < 0.05 (one-sample *t*-test) compared to a ratio of 1.

A-769662 binds the β1 subunit of AMPK, and there is evidence that A-769662 may act only on AMPK heterotrimers comprised of α_2_β_1_ subunits (Timmermans et al., 2014). Thus, to determine whether AMPK α_2_ and especially AMPK β_1_ subunits are expressed in arteries, the relative expression of these subunits was examined by comparing α_1_/α_2_ and β_1_/β_2_ expression ratios in muscular arteries (FA, EA, RA, and MA) and the large elastic CA using Western blot analysis. Expression of β_1_ was equal to expression of β_2_ in all arteries except FA, in which β_1_ expression was slightly but significantly less than β_2_ (**Figure 2A**). The expression of α_2_ was ∼2-fold that of α_1_ in all arteries except rabbit MA, which expressed approximately equal levels of α_1_ and α_2_ subunits (**Figure 2B**). The low α_1_/α_2_ expression of arterial muscle was not due to the inability of α_1_ antibody to detect α_1_ because, using the same antibodies and ratiometric analysis, we found that mouse ileum expressed nearly 100% α_1_, and conversely, that mouse extensor digitorum longus (EDL) expressed nearly 100% α_2_ (**Figure 2C**). Based on these data, there was no *a priori* reason to suspect that A-769662 would not be capable of inhibiting contractions induced in the rabbit arteries examined.

### Effect of A-769662 on Ang II-, KCl-, PE-, U-46619-, and VP-Induced CRCs in FA, EA, RA and MA

**FIGURE 3:**
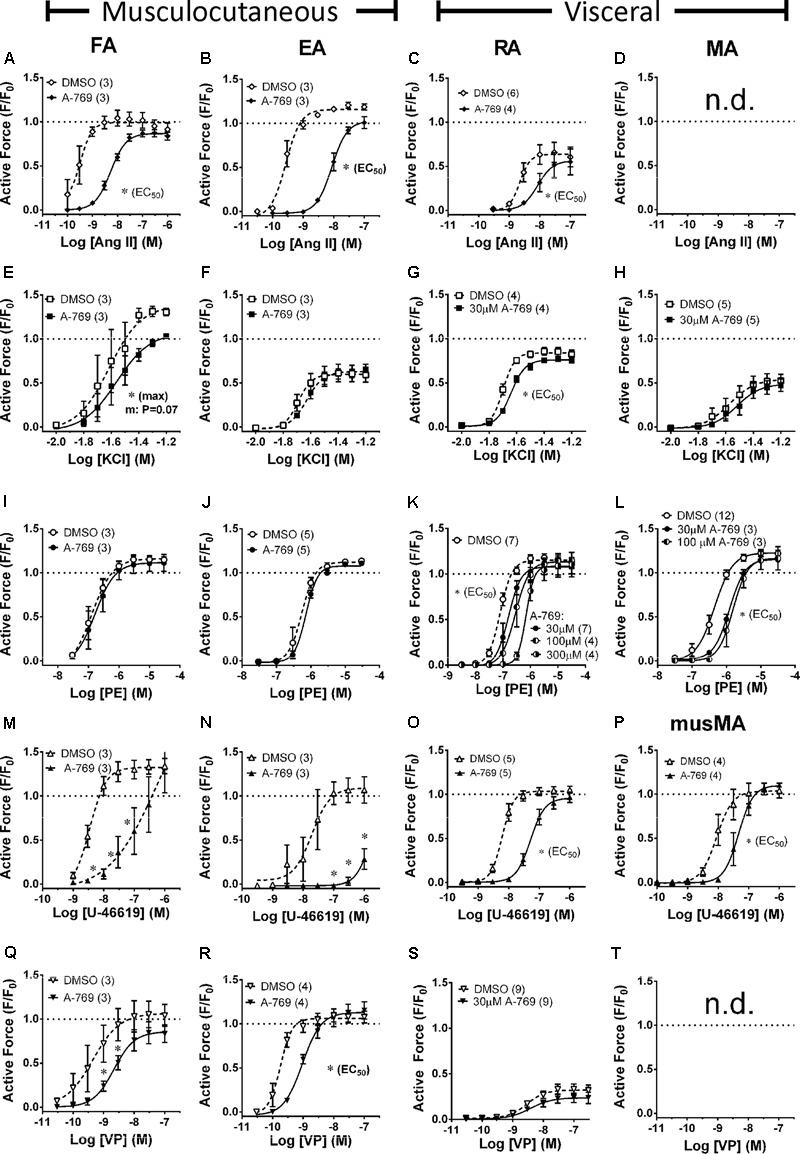
Effects of A-769662 (A-769, 30 μM unless indicated) on CRCs produced by Ang II (first row, **A–C**), KCl (second row, **E–H**), PE (third row, **I–L**), U-46619 (fourth row, **M–P**) and VP (last row, **Q–S**) in large (first column) and small (second column) musculocutanous arteries and large (third column) and small (last column) visceral arteries. FA, femoral artery; EA, epigastric artery; RA, renal artery; MA, mesenteric artery; musMA, mouse MA). Data are mean values ± SE; *n* values are in parentheses. ^∗^ is *P* < 0.05 (Student’s *t*-test) comparing the identified sigmoidal curve constant for groups A-769662 and vehicle control (DMSO). EC_50_, agonist concentration producing ½-maximum contraction. Max, calculated sigmoidal curve maximum tension. Data for A-769662 in **(D,I)** did not fit a sigmoidal curve, so A-769662 and control data at each agonist concentration were statistically compared. n.d., not determined.

**FIGURE 4:**
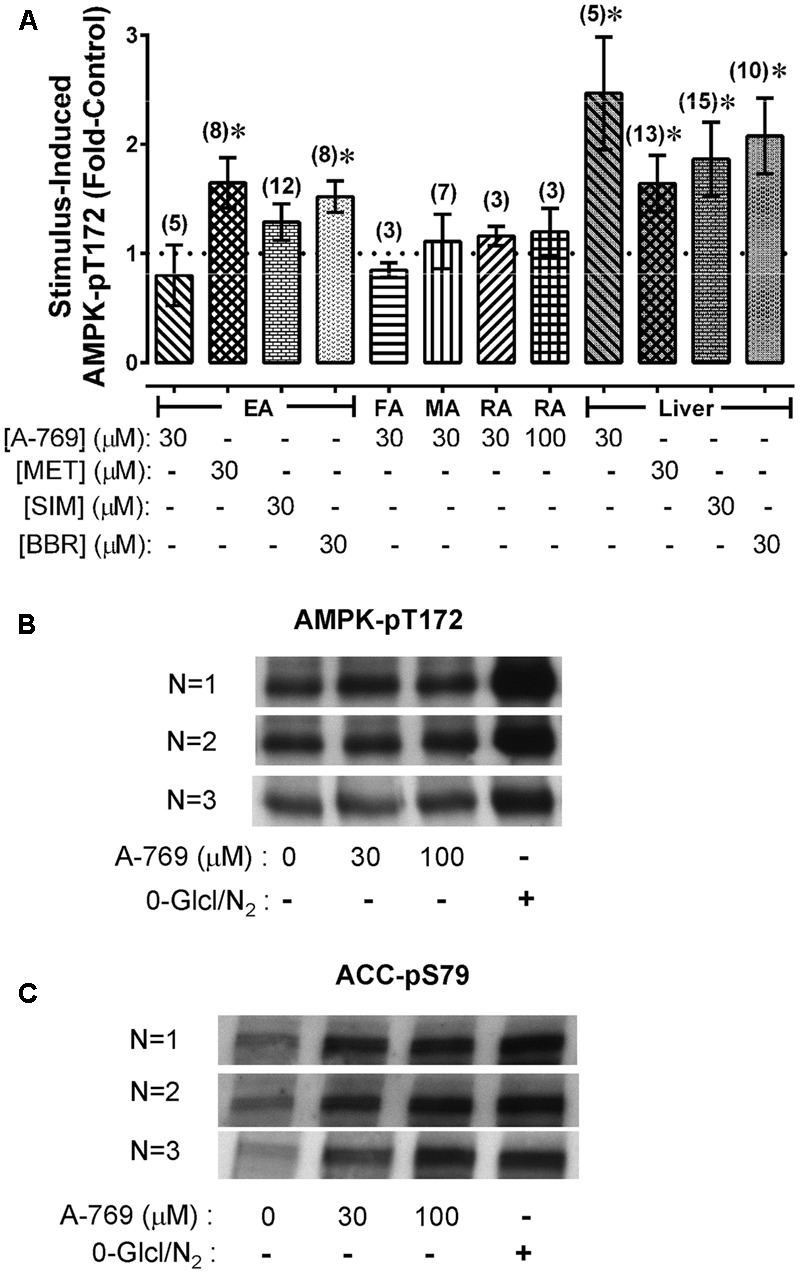
Average values of AMPK-pT172 in rabbit arteries and liver slices **(A)**, and example of Western blot data comparing the ability of A-769662 and a starvation protocol (0-Glc/N_2_) to cause an increase in AMPK-pT172 **(B)** and the AMPK substrate ACC-pS79 **(C)** in renal artery. Data in **(A)** are mean values ± SE; *n* values are in parentheses. ^∗^ is *P* < 0.05 one-sample *t*-test) compared to 1 (the control basal value). EA, epigastric artery; FA, femoral artery; MA, mesenteric artery; RA, renal artery.

**FIGURE 5:**
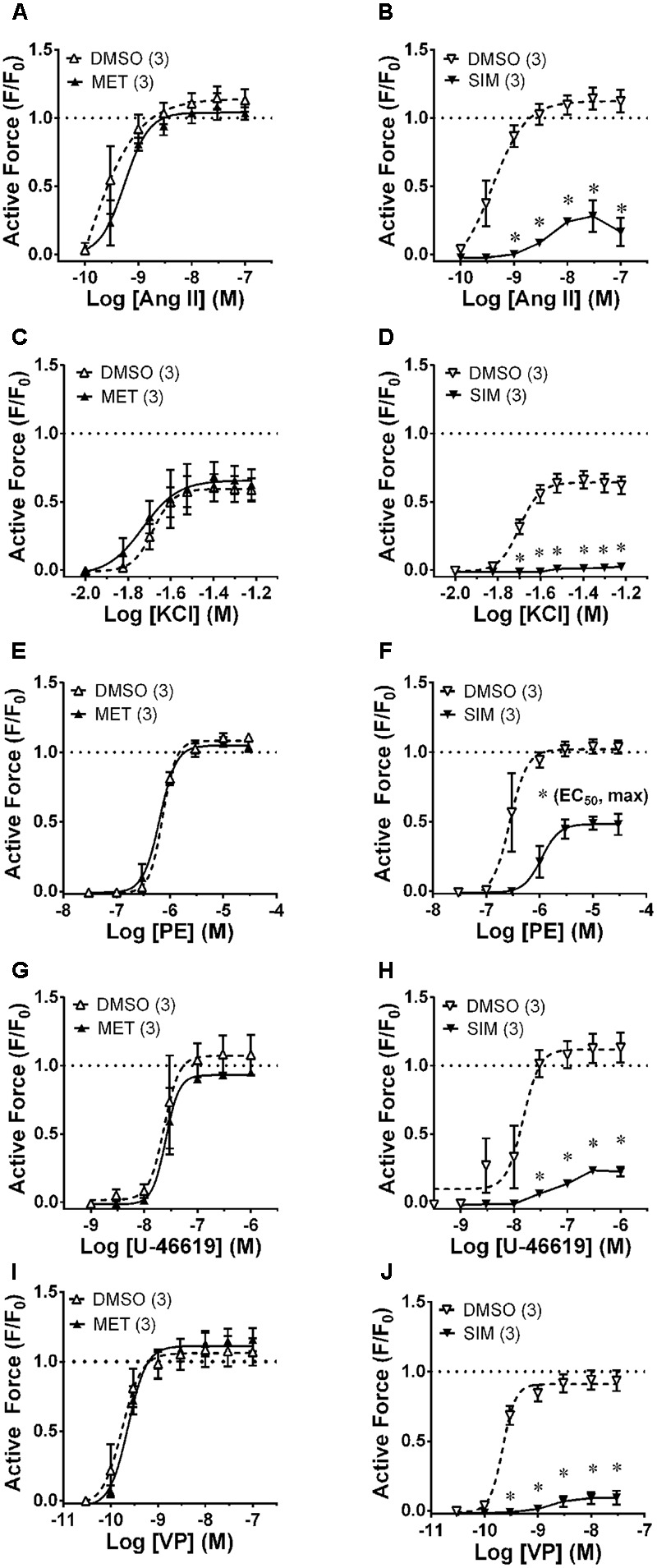
Effects of metformin (MET, 30 μM, first column) and simvastatin (SIM, 30 μM, second column) on CRCs produced by Ang II (first row, **A,B**), KCl (second row, **C,D**), PE (third row, **E,F**), U-46619 (fourth row, **G,H**) and VP (last row, **I,J**) in epigastric artery. Data are mean values ± SE; *n* values are in parentheses. ^∗^ is *P* < 0.05 (Student’s *t*-test) comparing A-769662 and vehicle control (DMSO) data at each agonist concentration and for **(F)**, comparing the identified sigmoidal curve constant for groups SIM and vehicle control (DMSO). EC_50_, agonist concentration producing ½-maximum contraction. Max, calculated sigmoidal curve maximum tension.

**FIGURE 6:**
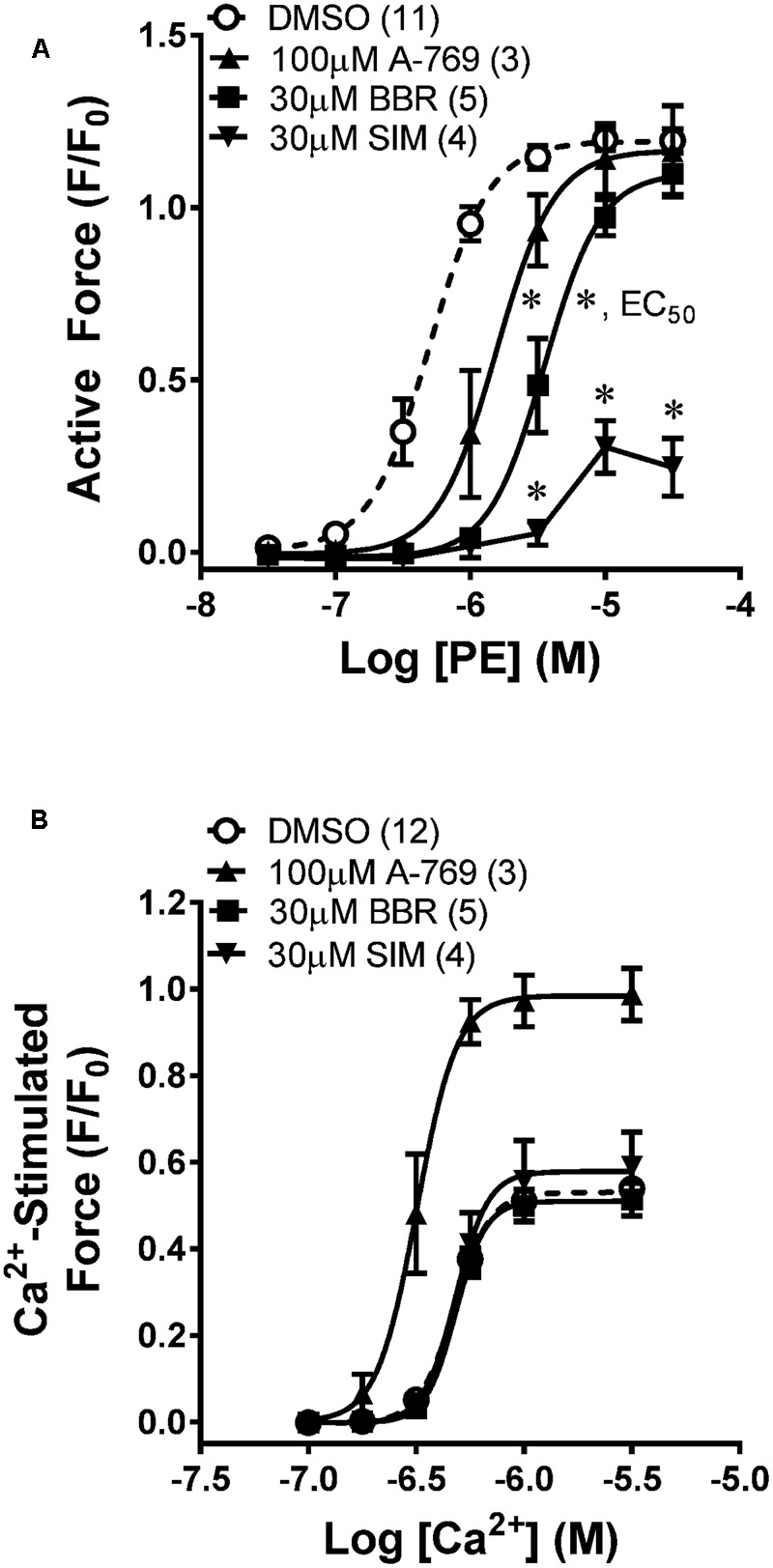
Effects of A-769662 (A-769), berberine (BBR), and simvastatin (SIM) on contractions induced by PE in intact mesenteric artery **(A)** and by Ca^2+^ in β-escin-permeabilized mesenteric artery **(B)**. Data are mean values ± SE; *n* values are in parentheses. ^∗^ is *P* < 0.025 (Student’s *t*-test with Bonferroni correction) comparing the EC_50_ values of A-769 and BBR groups to the vehicle control (DMSO). EC_50_, agonist concentration producing ½-maximum contraction. ^∗^ placed are SIM data points are *P* < 0.05 (Student’s *t*-test) comparing each data point from the SIM group to the control group.

**FIGURE 7:**
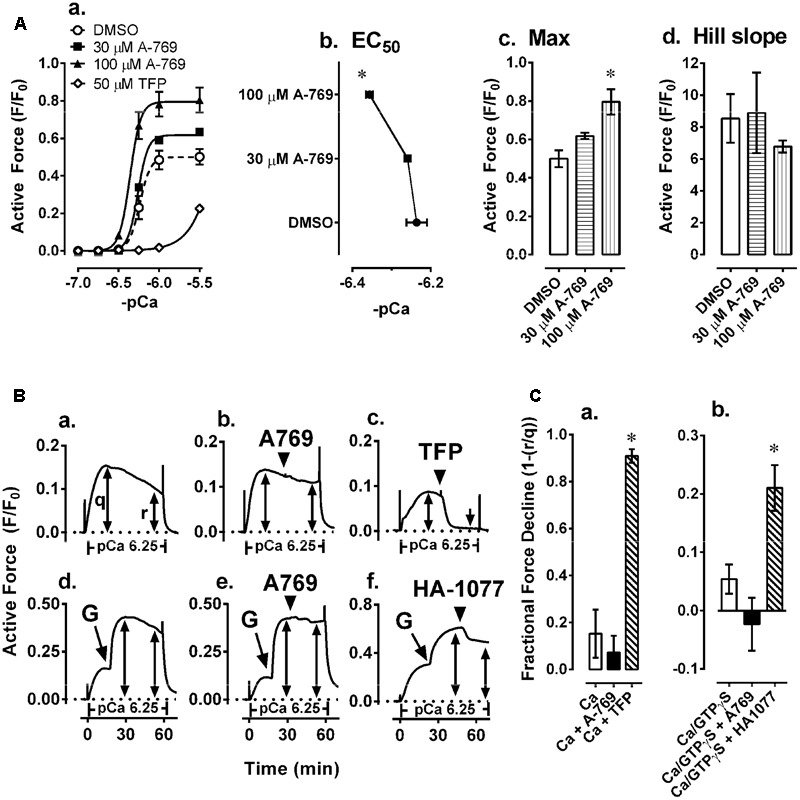
Effects of 30 and 100 μM A-769662 (A-769) and the calmodulin-MLKC inhibitor 50 μM trifluoperazine (TFP) on a Ca^2+^-induced CRC induced in β-escin-permeabilized mesenteric artery **(Aa)** and a comparison of the sigmoidal curve constants, EC_50_ **(Ab)**, maximum contraction, Max **(Ac)** and Hill slope **(Ad)**. Examples **(B)** and summary data **(C)** of effects of 30 μM A-769 **(Bb)** and 50 μM TFP **(Bc)** compared to control **(Ba)** on a Ca^2+^-induced contraction, and effects of 30 μM A-769 **(Be)** and 10 μM of the ROCK inhibitor HA-1077 **(Bf)** compared to control **(Bd)** on a GTPγS (“G” in panels **Bd–f** = GTPγS)-induced contraction induced in β-escin-permeabilized mesenteric artery. Data in **(A,C)** are mean values ± SE; *n* = 3–6. ^∗^ is *P* < 0.05 (Student’s *t*-test) compared to control. EC_50_ = agonist concentration producing ½-maximum contraction.

**FIGURE 8:**
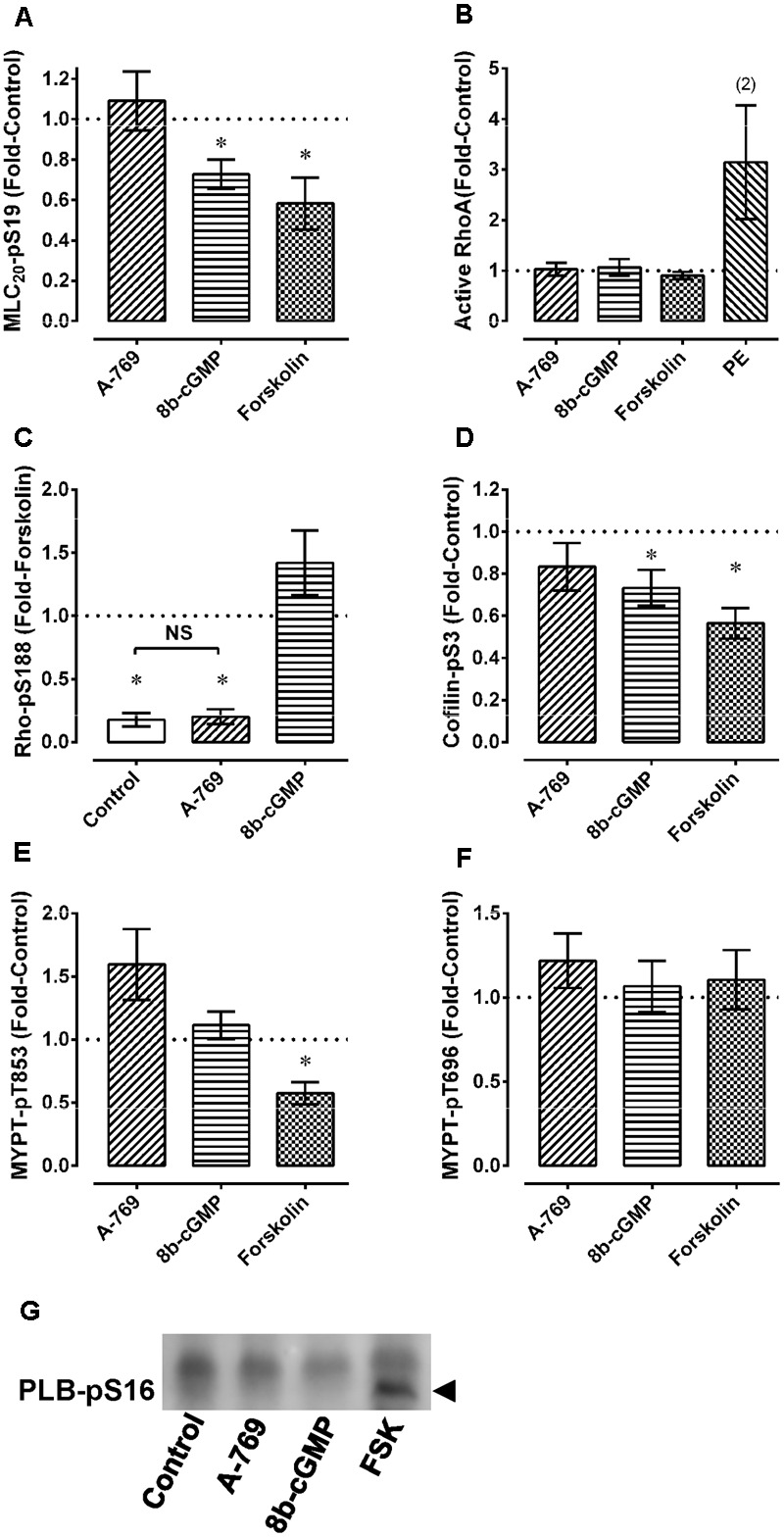
Effect of 30 μM A-769662 and other agents used as controls on basal levels of MLC-pS19 **(A)**, active rhoA **(B)**, rhoA-pS188 **(C)**, cofilin-pS3 **(D)**, MYPT1-pT853 **(E)** and MYPT1-pT696 **(F)** in femoral artery. An example of the effect of A-769662 (A-769) compared to the positive control forskolin (FSK) and to 8b-cGMP on PLB-pS16 is also shown (**G**, example from an *n* = 3). Data in **(A–F)** are mean values ± SE; *n* = 5–8 except where indicated. In **(A,D,E)**, ^∗^ is *P* < 0.05 (one-sample *t*-test) compared to the control value of 1. In **(C)**, ^∗^ is *P* < 0.025 (Bonferonni correction) compared to 1 (the control value) and NS, not significantly different. 8b-cGMP = 8 bromo-cGMP (100 μM); FSK = forskolin (10 μM); PE, phenylephrine (10 μM).

**FIGURE 9:**
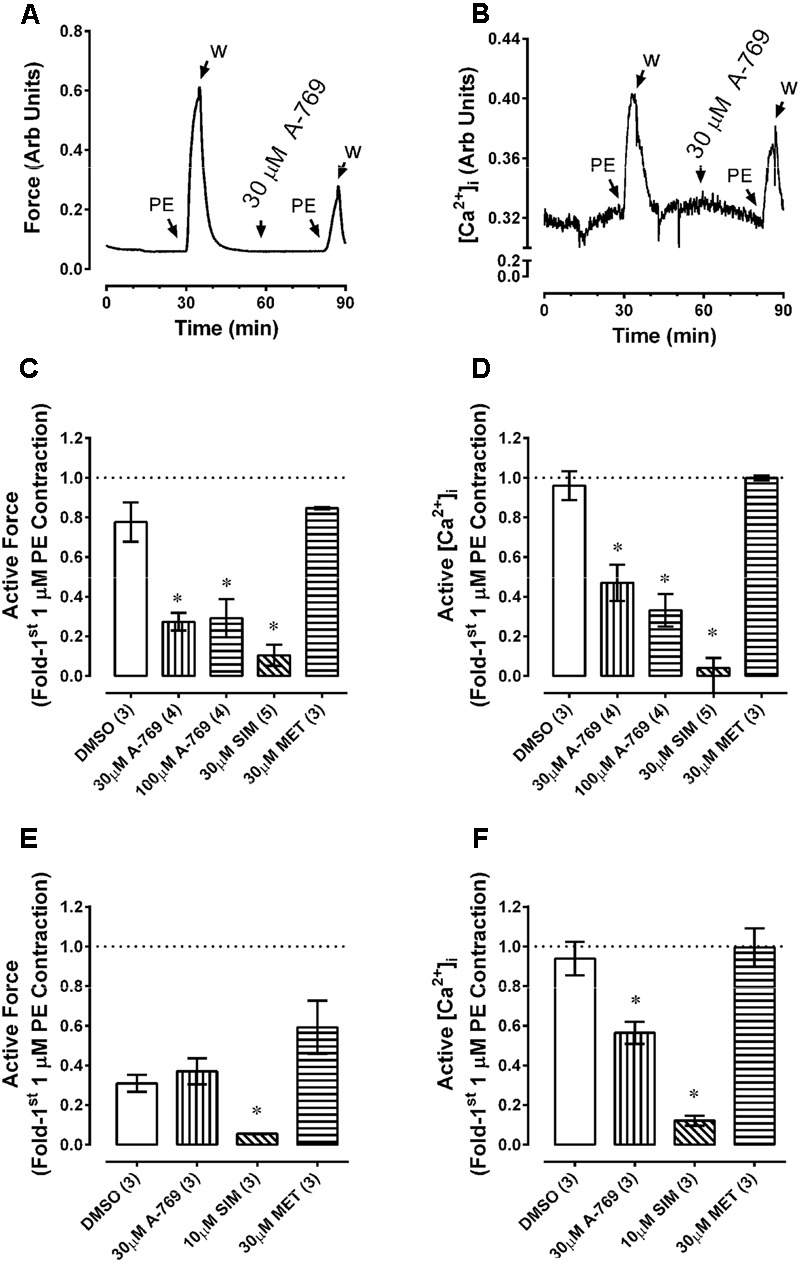
Effects of A-769662 (A-769), simvastatin (SIM) and metformin (MET) on contractile force and changes in intracellular free Ca^2+^ ([Ca^2+^]_i_) in fura-2-loaded renal **(A–D)** and mesenteric **(E,F)** arteries. Tissues were contracted with phenylephrine (PE) a first time to obtain control force and [Ca^2+^]_i_ data, then after stimulus washout exposed to either DMSO (vehicle control) or drug and stimulated a second time ∼30 min later with PE. Data are example tracings of force **(A)** and [Ca^2+^]_i_ **(B)**, and summary data (mean force values ± SE; force, **(C,E)**, and [Ca^2+^]_i_, **(D,F)**; *n* = 3–5) expressed as the second response normalized to the first response. ^∗^ is *P* < 0.05 (ANOVA and Dunnett’s) compared to DMSO control.

**FIGURE 10:**
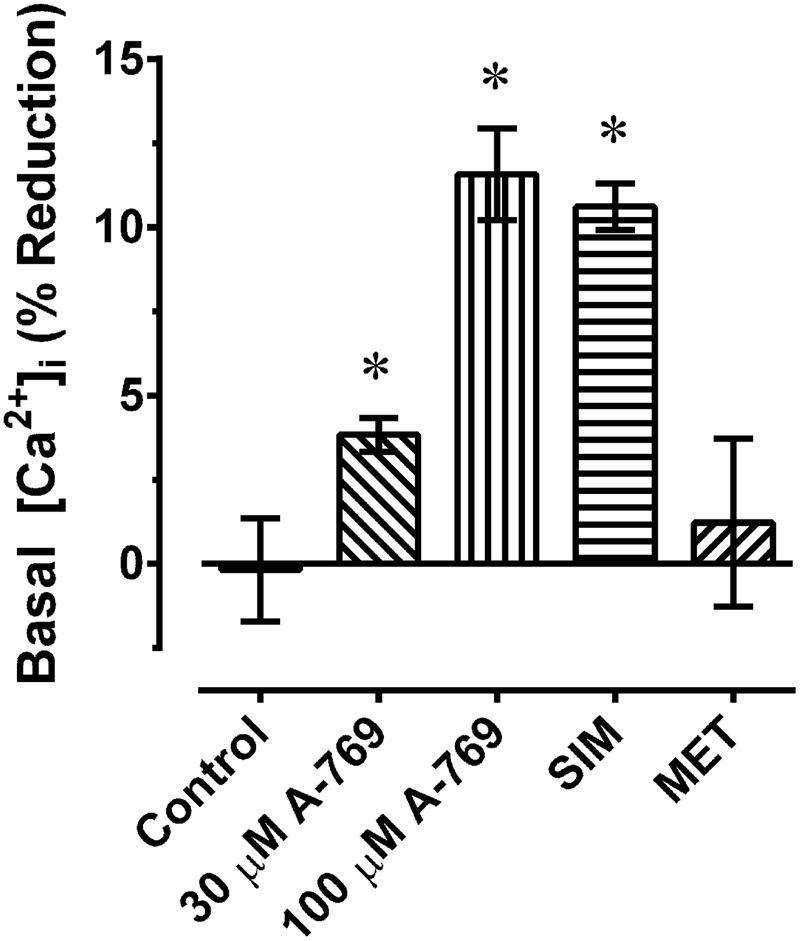
Percent reduction in basal [Ca^2+^]_i_ induced by 30 and 100 μM A-769662 (A-769), simvastatin (SIM, 30 μM) and metformin (MET, 30 μM) in renal artery. Data are mean values ± SE; *n* = 3–5. ^∗^ is *P* < 0.05 (one-sample *t*-test) compared to zero.

**FIGURE 11:**
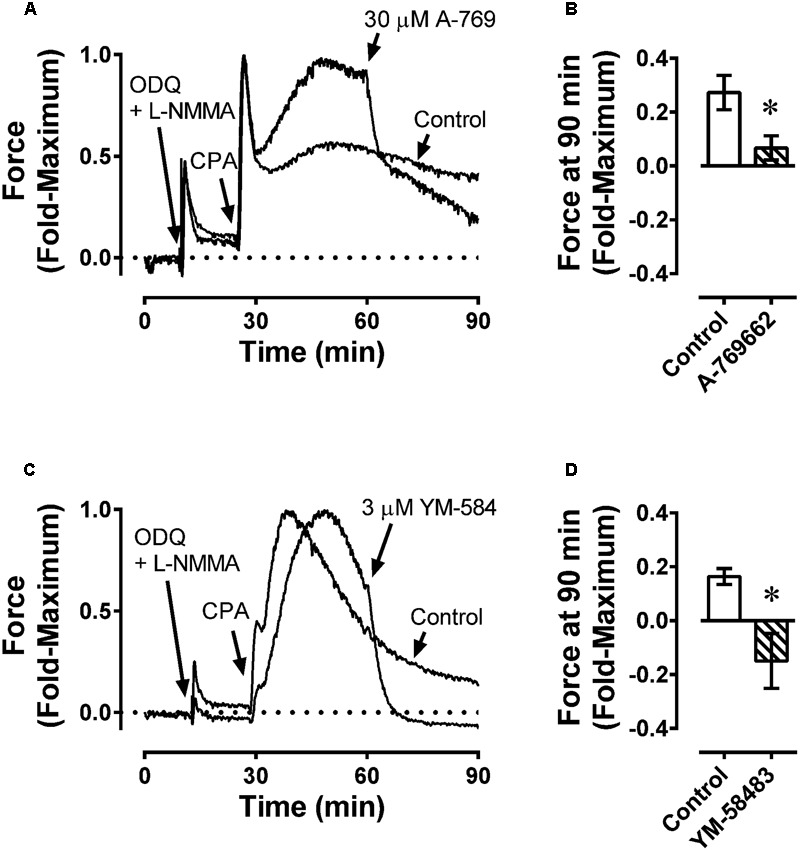
Example force tracings **(A,C)** and summary data **(B,D)** showing effect of A-769662 (30 μM A-769, **A,B**) and the selective store-operated Ca^2+^ channel blocker YM-58483 (3 μM YM-584, **C,D**) on contraction produced by the sarcoplasmic reticulum Ca^2+^ store depleting agent, cyclopiazonic acid (10 μM CPA) in femoral artery. ODQ and L-NMMA were administered first to inhibit NO-dependent increases in VSM cGMP, which caused a weak contraction. Data in **(B,D)** are mean values ± SE; *n* = 4. ^∗^ is *P* < 0.05 (Student’s t-test) compared to vehicle control.

Once activated, AMPK is proposed to cause relaxation (inhibition of contraction) of large elastic arteries (e.g., aorta) and not smaller muscular arteries by an endothelium-independent mechanism (Goirand et al., 2007; Davis et al., 2012). However, A-769662 was shown to inhibit small muscular arteries of the mouse and hamster (Schneider et al., 2015). Thus, the ability of A-769662 to inhibit both large and small muscular arteries was examined to determine whether A-769662 can effectively relax muscular arteries known to display functional differences. In particular, this experiment analyzed the ability of A-769662 to inhibit contractions induced by Ang II (**Figures 3A–C**), KCl (**Figures 3E–H**), PE (**Figures 3I–L**), U-46619 (**Figures 3M–P**), and VP (**Figures 3Q–S**) in several different muscular arteries, including large musculocutaneous (FA, **Figure 3**, first column) and visceral (RA, **Figure 3**, third column) arteries, and small musculocutaneous (EA, **Figure 3**, second column) and visceral (MA, **Figure 3**, fourth column) arteries. In both large and small musculocutaneous arteries, A-769662 strongly inhibited contractions induced by Ang II and U-46619, and modestly but significantly inhibited contractions induced by VP. By contrast, the weak VP contractile response induced in the visceral RA was not inhibited by A-769662. Also by contrast, A-769662 had no effect on contractions induced by PE in musculocutaneous arteries, but did cause a reduction in the contractile potency of visceral arteries. In visceral arteries, 30 μM A-769662 did not reduce the maximum efficacy of contraction. This was true even when employing higher A-769662 concentrations (100 and 300 μM, **Figures 3K,L**). Moreover, A-769662 only weakly inhibited KCl-induced contractions in the larger FA and RA, and had no effect on KCl-induced contractions in the smaller EA and MA.

In summary, A-769662 induced a broad range of relaxant effects, from very strong inhibition to no inhibition of muscular arteries, and in nearly every case where A-769662 exerted inhibition of contraction, higher concentrations of stimulus overcame the inhibition. In short, A-769662 displayed differential inhibitory activity when considering (1) stimulus-type, (2) musculocutaneous vs. visceral arteries, and (3) large vs. small muscular arteries. Together, these data do not support a mechanism of A-769662-induced inhibition of contraction that involves a signaling mechanism downstream from and common to all of the contractile stimuli and all of the vascular segments examined, such as MLCK and ROCK.

To determine whether 30 μM A-769662 could cause an NO-cGMP-mediated relaxation in EA, tissues not denuded of endothelium and not treated with ODQ nor L-NMMA were contracted in a step-wise fashion with increasing PE concentrations. CRCs produced in the presence of A-769662 were not different than control data, indicating that A-769662 did not cause relaxation of a PE-induced contraction by a nitric oxide-cGMP-dependent pathway in EA (*n* = 3, data not shown).

### Effect of A-769662 and a Glucose- and O_2_-Free Solution (Starved) on Indices of AMPK Activation

Our data show that A-769662 caused strong relaxation of certain arteries. To determine whether arterial relaxation induced by A-769662 can be linked to an increase in AMPK-pT172, EA, FA, MA and RA were treated for 60 min with A-769662, and the level of AMPK-pT172 was measured and compared to control, untreated, tissues. A-769662 did not cause an increase AMPK-pT172 in any artery examined (**Figure 4A**). By contrast, 30 μM A-769662 induced a ∼2-fold increase in AMPK-pT172 in liver slices (**Figure 4A**). Two other known AMPK activators, MET and BBR, increased AMPK-pT172 above the basal level in both EA and liver, and a third known AMPK activator, SIM, increased AMPK-pT172 in liver but not in EA (**Figure 4A**). Although 30 and 100 μM A-769662 did not increase AMPK-pT172 in RA, starvation (no glucose added to the buffer and O_2_ depletion by vigorous bubbling with 100% N_2_) (Smith et al., 2017) caused a 2.2 ± 0.4-fold increase (**Figure 4B**, *n* = 3). Notably, both A-769662 and starvation caused a ∼2-fold increase in the downstream AMPK substrate ACC-pS79 in RA (**Figure 4C**), suggesting that A-769662 may activate AMPK without elevating the degree of AMPK-pT172 in rabbit artery.

### Effect of MET and SIM on Ang II-, KCl-, PE-, U-46619-, and VP-Induced CRCs in EA

To compare the relaxant effects of A-769662 with MET and SIM, EA was contracted in the presence and absence of 30 μM MET (**Figure 5**, first column) and 30 μM SIM (**Figure 5**, second column). A-769662, MET and SIM displayed differential effects on contraction of the small musculoskeletal muscular artery EA, ranging from no inhibition to strong inhibition [compare **Figure 3** column 2 (EA) with **Figure 5**]. Notably, MET, which caused an increase in AMPK-pT172 in EA (see **Figure 4A**), had no effect on contractions induced by any stimulus examined (**Figure 5**, first column), and SIM that did not elevate AMPK-pT172 (see **Figure 4A**) caused inhibition of contractions induced by all of the stimuli tested (**Figure 5**, second column).

### Effect of A-769662 on β-Escin-Permeabilized Artery and on Phosphoprotein Indices of Contractile Protein Regulation in MA

Chemical permeabilization abolishes plasma membrane Ca^2+^ regulation and permits access to the cytosol so that the cytosolic Ca^2+^ level can be directly controlled. To determine whether A-769662 can cause relaxation independently of an effect on plasma membrane Ca^2+^ regulation, MA was permeabilized with β-escin and a Ca^2+^ CRC was generated in the presence and absence of 100 μM A-769662. Whereas 100 μM A-769662 reduced the potency of a PE CRC in intact MA (**Figure 6A**), in β-escin-permeabilized MA, 100 μM A-769662 *increased* the potency and maximum efficacy of a Ca^2+^ CRC (**Figures 6B, 7A**). For comparison, BBR and SIM were also examined. In intact MA, 30 μM BBR, like 100 μM A-769662, reduced the potency of a PE CRC, and 30 μM SIM dramatically reduced the maximum efficacy (**Figure 6A**). However, neither BBR nor SIM had an effect on a Ca^2+^ CRC in permeabilized MA (**Figure 6B**). Together, these data indicate that when the cytosolic Ca^2+^ level is “clamped” at discrete levels (Ca^2+^-CRC in permeabilized muscle), A-769662, BBR and SIM did not cause VSM relaxation, suggesting that the relaxation of non-permeabilized (plasma membrane-intact) muscle by these agents was due to an alteration in Ca^2+^ regulation at the plasma membrane and not to signaling pathways down-stream from Ca^2+^, such as MLCK, or independent of Ca^2+^, such as ROCK.

As a positive control, the calmodulin and thus MLCK blocker TFP strongly inhibited Ca^2+^-induced contractions in permeabilized MA as expected (**Figures 7Aa,Bc,Ca**). To further test the hypothesis that the A-769662-induced relaxation of contraction was not due to an inhibition of signaling systems downstream from, or independent of, Ca^2+^, permeabilized MA was contracted submaximally with Ca^2+^ at pCa 6.25, then contracted further by addition of GPTγS (“G” in **Figures 7Bd–f**) to cause Ca^2+^ sensitization by activation of ROCK. A-769662 had no effect on the contraction induced by Ca^2+^ (**Figures 7Bb,Ca**) compared to control (**Figure 7Ba**) or that induced by GTPγS (**Figure 7Be**, “G”, **Figure 7Cb**) compared to control (**Figure 7Bd**). As expected, TFP strongly inhibited the Ca^2+^-induced contraction (**Figures 7Bc,Ca**), and the ROCK inhibitor H-1077 depressed the contraction induced by GTPγS (**Figure 7Bf**, “G”, **Figure 7Cb**). Moreover, A-769662 had no effect on the basal phosphorylation levels of MLC (**Figure 8A**), rhoA (**Figure 8C**), cofilin (**Figure 8D**), and MYPT1 (**Figures 8E,F**), and did not reduce the degree of active rhoA (**Figure 8B**) in FA. Likewise, A-769662 did not cause an increase in PLB-pS16 in FA as did FSK, a positive control (**Figure 8G**). Together, these data support the previous experiments suggesting that A-769662 did not cause inhibition of contraction by inhibiting MLCK or ROCK.

### Effect of A-769662 on [Ca^2+^]_i_ in Visceral Arteries

Results from chemically permeabilized artery support the hypothesis that the A-769662-induced relaxation of plasma membrane-intact artery was due to an effect on plasma membrane Ca^2+^ regulation. To test this hypothesis, the effect of A-769662 on cytosolic free Ca^2+^ was examined in fura-2-loaded artery. Concomitant with its ability to inhibit contraction (**Figure 9A**), 30 μM A-769662 inhibited the ability of a submaximum concentration of PE to induce a strong increase in [Ca^2+^]_i_ (**Figure 9B**). This was true both for RA (**Figures 9A–D**) and MA (**Figures 9E,F**). For a comparison, SIM also caused a strong inhibition of PE-induced contraction and increase in [Ca^2+^]_i_, whereas MET did not (**Figures 9C–F**). Notably, A-769662 and SIM, and not MET, produced an immediate reduction in the basal level of [Ca^2+^]_i_ (**Figure 10**, and see **Figure 9B**). The strong reduction in force during a second contraction (**Figure 9E**, DMSO) that did not correlate with a reduction in [Ca^2+^]_i_ (**Figure 9F**, DMSO) in MA was likely due to receptor-activation induced desensitization (arterial memory) (Ratz, 1995, 1999).

### Effect of A-769662 on CPA-Induced Contraction in FA

Data from the fura-2-loaded artery experiments suggest that A-769662 caused relaxation by inhibiting Ca^2+^ mobilization. To determine whether inhibition of store-operated Ca^2+^ entry played a role in A-769662-induced relaxation, the ability of A-769662 to relax a CPA-induced contraction in FA was assessed. Inhibition of the SERCA pump by CPA causes the release of sarcoplasmic reticulum Ca^2+^ stores which, in turn, activates Ca^2+^ entry through store-operated Ca^2+^ channels located in the plasma membrane. In FA, 10 μM CPA caused an initial strong contraction that declined to a weaker steady-state response (**Figures 11A,C**, Control). A-769662 added during the tonic phase of a CPA-induced contraction (∼30 after CPA addition) caused an immediate relaxation (**Figure 11A**, 30 μM A-769), reducing the steady-state response at 90 min from ∼25% of the peak contraction to ∼10% (**Figure 11B**). By comparison, the selective store-operated Ca^2+^ channel inhibitor YM-58483 (a.k.a, BTP2) (Harper and Poole, 2011) also caused an immediate relaxation (**Figure 11C**, 3 μM YM-584) and reduced the steady-state response to ∼-10% (**Figure 11D**). That is, YM-58483 reduced force to a value less than the resting value, indicating that FA displayed a small degree of active tone. Together, these data suggest that A-769662 caused relaxation, in part, by immediate inhibition of store-operated Ca^2+^ channels.

## Discussion

Several studies support the hypothesis that AMPK activation directly inhibits several contractile protein regulatory systems causing VSM relaxation, and that the AMPK activator A-769662 acts as a vascular relaxant agent by activating AMPK (Goirand et al., 2007; Horman et al., 2008; Ford and Rush, 2011; Schneider et al., 2015). The primary goal of the present study was to further investigate this hypothesis. Based on assessments of the ability of A-769662 to inhibit contractions induced in intact and permeabilized arteries, to inhibit the basal levels of phosphorylation of contractile protein regulatory proteins, and to inhibit basal and stimulated [Ca^2+^]_i_, our results do not support the hypothesis that A-769662 causes contraction inhibition by activation of AMPK, or that AMPK activation is both necessary and sufficient to inhibit contraction. Rather, our working model is that A-769662, like SIM (Bergdahl et al., 2003), caused VSM relaxation by altering mechanisms related to Ca^2+^ mobilization independently of an increase in AMPK activation.

In a cell-free kinase assay, 10 μM A-769662 inhibits smooth muscle MLCK by ∼23%, ROCK by ∼16%, and PKCα by ∼12% (Goransson et al., 2007). Moreover, active AMPK has been proposed to inhibit smooth muscle contraction by inhibition of MLCK (Horman et al., 2008), ROCK (Wang et al., 2011), and PKC (Davis et al., 2012). Thus, A-769662 may cause VSM relaxation by directly inhibiting key kinases responsible for contraction, as well as by activating AMPK that, in turn, inhibits these kinases. Both 1 μM GF-109203X and 1 μM HA-1152, inhibitors of, respectively, PKC and ROCK, cause a rightward shift of the PE CRC in rabbit EA, indicating that PE-induced contractions in this artery are dependent, in part, on PKC and ROCK (Ratz, 2016). Thus, if A-769662 can cause relaxation by inhibition of ROCK and PKC, then A-769662 would be expected to suppress PE-induced contractions. Likewise, A-769662 would be expected to inhibit Ca^2+^-activated contraction of β-escin-permeabilized VSM because ROCK and PKC inhibitors attenuate this response (Clelland et al., 2011). However, a PE-induced contraction of EA was entirely resistant to A-769662. In β-escin-permeabilized artery, A-769662 did not inhibit Ca^2+^-activated contraction, nor did A-769662 cause relaxation of a GTPγS-induced contraction that was attenuated by the ROCK inhibitor HA-1077. Moreover, A-769662 had no effect on the basal level of MYPT-pT853, a ROCK substrate, on cofilin-pS3, a ROCK-LIMK substrate, or on rhoA activity. Together, these data suggest that neither ROCK nor PKC were A-769662 targets involved in causing inhibition of VSM contraction.

In large (RA) and small (MA) rabbit muscular visceral arteries, 30 and 100 μM A-769662 reduced the basal level of [Ca^2+^]_i_ and inhibited a PE-induced contraction by reducing the PE-induced increase in [Ca^2+^]_i_. We did not identify the precise mechanism causing [Ca^2+^]_i_ inhibition. However, the absence of an increase in PLB phosphorylation by A-769662 at the site stimulated by both cAMP-dependent protein kinase and AMPK suggests that we can rule out activation of SERCA that would lower [Ca^2+^]_i_, a mechanism proposed for A-769662-induced relaxation of mouse resistance arteries (Schneider et al., 2015). Moreover, our data do not support inhibition by A-769662 of L-type voltage-operated Ca^2+^ channels because A-769662 was a relatively poor inhibitor of KCl-induced contractions that are largely dependent on activation of these channels. We found that A-769662 inhibited the steady-state contraction induced by CPA in FA in a manner similar to the selective store-operated Ca^2+^ channel inhibitor, YM-58483 (Harper and Poole, 2011). Notably, YM-58483 was shown to inhibit STIM-1/Orai-1 induced store-operated Ca^2+^ entry in ethanol-treated rats (Souza Bomfim et al., 2017). Thus, we propose that one mechanism by which A-769662 causes VSM relaxation is via inhibition of store-operated Ca^2+^ entry. However, we cannot rule out the possibility that relaxation was induced by VSM hyperpolarization, because A-769662 has been shown to indirectly activate VSM potassium channels by causing the releasing of a hyperpolarizing factor from perivascular fat of rat MA (Weston et al., 2013).

The antidiabetic drug MET and lipid-lowering statins such as SIM activate AMPK in several cell types (Sun et al., 2006; Hawley et al., 2010; Hardie, 2013). SIM also has been shown to cause VSM relaxation by inhibiting Ca^2+^ entry (Alvarez de Sotomayor et al., 2001; Bergdahl et al., 2003). In EA, MET did, but SIM did not, cause an increase in AMPK-pT172. Notably, MET did not inhibit contractions induced by any stimulus examined in this artery, and SIM caused strong inhibition of all stimuli. These data indicate that an increase in AMPK-pT172 will not necessarily cause VSM relaxation. Interestingly, both SIM and A-769662 did not cause an increase in AMPK-pT172 and yet inhibited basal and stimulated [Ca^2+^]_i_. SIM caused insurmountable inhibition of contractions (see **Figure 5**, second column), whereas A-769662-induced inhibition was surmountable under all conditions except a KCl-induced CRC in FA (see **Figure 2**). Thus, the mechanism by which SIM and A-769662 inhibited Ca^2+^ mobilization are likely different.

In summary, based on the observations that an increase in AMPK-pT172 did not necessarily cause VSM relaxation, and that A-769662 did not increase AMPK-pT172 above the basal level, our working model is that A-769662 may have acted independently of an increase in AMPK-pT172 to inhibit basal [Ca^2+^]_i_ and stimulus-induced increases in [Ca^2+^]_i_ which, in turn, reduced the strength of VSM contraction. Although contraction inhibition was produced in both large and small musculocutaneous and visceral arteries, the degree of inhibition displayed a dependency on both artery- and stimulus-type. Specifically, A-769662 strongly inhibited contractions induced by Ang II and U-46619 in all arteries examined, modestly inhibited contractions induced by PE in visceral arteries, and did not inhibit contractions induced by PE in musculocutaneous arteries. KCl-induced contractions were inhibited weakly in the larger FA and RA, and not inhibited in the smaller EA and MA. Thus, the effect of A-769662 may be dependent on the expression levels in different artery segments of a specific signaling systems regulating [Ca^2+^]_i_. Although A-769662 did not cause an increase in arterial AMPK-pT172, it did cause an increase in phosphorylation of the AMPK substrate ACC. Thus, although our study supports a model by which A-769662 caused contraction inhibition by an AMPK-independent mechanism, our data cannot rule out the possibility that A-769662 enhanced AMPK activity independently of an increase in AMPK-pT172 (Goransson et al., 2007; Foretz et al., 2010), and that activated AMPK exerted an artery- and stimulus-selective inhibition of Ca^2+^ mobilization.

## Author Contributions

YH was responsible for assisting in protocol development, data acquisition and analysis and in assisting in the drafting of the article. CS, GC, BS, and AM were responsible for data acquisition. RB was responsible for revising the article for intellectual content. PR was responsible for project concept, protocol development, data analysis and interpretation, drafting the article and revising the article for intellectual content, and for final approval of the completed article.

## Conflict of Interest Statement

The authors declare that the research was conducted in the absence of any commercial or financial relationships that could be construed as a potential conflict of interest.

## Footnotes

1 http://imagej.nih.gov/ij/

## Abbreviations

ACC-pS79: acetyl-CoA carboxylase serine 79 phosphorylation
AMPK: 5′-adenosine monophosphate kinase
AMPK-pT172: AMPK threonine-172 phosphorylation
Ang II: angiotensin II
BBR: berberine
CRC: concentration-response curve
EA: epigastric artery
FA: femoral artery
L-NMMA: L-N^G^-monomethyl arginine
MA: mesenteric artery
MET: metformin
MLC-pS19: myosin regulatory light chain serine 19 phosphorylation
MYPT1: myosin phosphatase targeting/regulatory subunit 1
ODQ: 1H-[1,2,4]oxadiazolo[4,3-a]quinoxalin-1-one
PE: phenylephrine
PKC: protein kinase C
PLB-pS16: phospholamban serine 16 phosphorylation
RA: renal artery
ROCK: rho kinase
SIM: simvastatin
VP: vasopressin
